# Identification of misdiagnosis by deep neural networks on a histopathologic review of breast cancer lymph node metastases

**DOI:** 10.1038/s41598-022-17606-0

**Published:** 2022-08-05

**Authors:** Cancan Chen, Shan Zheng, Lei Guo, Xuebing Yang, Yan Song, Zhuo Li, Yanwu Zhu, Xiaoqi Liu, Qingzhuang Li, Huijuan Zhang, Ning Feng, Zuxuan Zhao, Tinglin Qiu, Jun Du, Qiang Guo, Wensheng Zhang, Wenzhao Shi, Jianhui Ma, Fenglong Sun

**Affiliations:** 1 Digital Health China Technologies Corporation Limited, Beijing, 100080 China; 2 Department of Pathology, National Cancer Center/National Clinical Research Center for Cancer/Cancer Hospital, Chinses Academy of Medical Sciences and Peking Union Medical College, Beijing, 100021 China; 3 Department of Pathology, National Cancer Center/National Clinical Research Center for Cancer/Hebei Cancer Hospital, Chinese Academy of Medical Sciences, Langfang, 065001 China; 4 Research Center of Precision Sensing and Control, Institute of Automation, Chinese Academy of Sciences, Beijing, 100190 China; 5 Department of Medical Affairs, National Cancer Center/National Clinical Research Center for Cancer/Cancer Hospital, Chinese Academy of Medical Sciences and Peking Union Medical College, Beijing, 100021 China; 6 Department of Academic Research, National Cancer Center/National Clinical Research Center for Cancer/Cancer Hospital, Chinese Academy of Medical Sciences and Peking Union Medical College, Beijing, 100021 China; 7 Department of Big Data, National Cancer Center/National Clinical Research Center for Cancer/Cancer Hospital, Chinese Academy of Medical Sciences and Peking Union Medical College, Beijing, 100021 China; 8 Department of Urology, National Cancer Center/National Clinical Research Center for Cancer/Cancer Hospital, Chinese Academy of Medical Sciences and Peking Union Medical College, Beijing, 100021 China

**Keywords:** Image processing, Machine learning

## Abstract

The frozen section (FS) diagnoses of pathology experts are used in China to determine whether sentinel lymph nodes of breast cancer have metastasis during operation. Direct implementation of a deep neural network (DNN) in clinical practice may be hindered by misdiagnosis of the algorithm, which affects a patient's treatment decision. In this study, we first obtained the prediction result of the commonly used patch-DNN, then we present a relative risk classification and regression tree (RRCART) to identify the misdiagnosed whole-slide images (WSIs) and recommend them to be reviewed by pathologists. Applying this framework to 2362 WSIs of breast cancer lymph node metastasis, test on frozen section results in the mean area under the curve (AUC) reached 0.9851. However, the mean misdiagnosis rate (0.0248), was significantly higher than the pathologists’ misdiagnosis rate (*p* < 0.01). The RRCART distinguished more than 80% of the WSIs as a high-accuracy group with an average accuracy reached to 0.995, but the difference with the pathologists’ performance was not significant (*p* > 0.01). However, the other low-accuracy group included most of the misdiagnoses of DNN models. Our research shows that the misdiagnosis from deep learning model can be further enriched by our method, and that the low-accuracy WSIs must be selected for pathologists to review and the high-accuracy ones may be ready for pathologists to give diagnostic reports.

## Introduction

Deep neural networks (DNNs) have shown a great progress and promise in image classification^1^. For example, the inception network^2^, ResNet^3,4^ and Xception^5^ perform similar to or better than humans in the ImageNet (http://www.image-net.org/) classification task. The Cancer Metastases in Lymph Nodes Challenge 2016 (CAMELYON16, https://camelyon16.grand-challenge.org/) has greatly promoted the implementation of DNNs for whole slide images (WSIs) in cancer histopathology and the gradual development of the patch-DNN method^6,7^: at the patch level, a trained DNN model is used to generate a heatmap, while at the WSI level, the results of diagnosis are directly given through max-pooling^8^, or features for heatmaps are extracted, and then the diagnosis results for the WSIs are generated by different machine-learning classifiers^9,10^. DNN training tricks^8^ and architecture updates^11,12^ are also dependent on the patch-DNN. The encouraging results have been obtained for the prostate (AUC, 0.986), skin cancer basal cell carcinoma (AUC, 0.986), and axillary lymph nodes (AUC, 0.965)^10^, another study of prostate cancer with overall accuracy of 0.973^13^, a bladder cancer dataset (AUC, 0.95)^12^, axillary lymph node data from CAMELYON16 (AUC > 0.99)^6^, gastric cancer (sensitivity, near 100%, specificity, 80.6%)^14^ and lung adenocarcinoma and squamous cell carcinoma (AUC 0.9594 and 0.9414)^15^ , cervical cancer (93.5% Specificity and 95.1% Sensitivity)^16^. The patch-DNN, acting as a diagnostic assistant tool, can significantly improve a pathologist’s work efficiency^17^. However, misdiagnosis is an inevitable limitation of the algorithm and hinder the broad application of this assistant tool in pathology.

There are two strategies to improve the model misdiagnosis issue: increasing accuracy and decreasing misclassification. To overcome this problem, most literatures^6,8–13,15,16^ were based on improving the classification accuracy performance of patch-DNNs, but the encouraging results achieved in recent years have reached or are close to its ceiling. To decrease misclassification, precious work applied screening method for enrichment of malignant WSIs and disregarding the benign ones by adjusting probability threshold, this method achieved the removal of more than 75% of the benign from the workload of a pathologist without reducing in sensitivity on prostate cancer^10^, similarly, Song et al. achieved a sensitivity near 100% and an average specificity of 80.6% on gastric cancer^14^. however, this method cannot obtain good generalizability to be widely used in different dataset. In this study, we used a new machine learning method based on the results of patch-DNN prediction to reduce the error rate. We presented a relative risk classification and regression tree (RRCART) to identify the misdiagnosis made by the commonly used patch-DNN and evaluated the identification performance by a Poisson distribution estimated from pathologists’ misdiagnosis data. To develop the new method and avoid blind application without obtaining the slightest insight into the clinical problem, we chose the diagnosis of sentinel lymph node (SLN) metastasis of breast cancer based on frozen sections (FSs) as a practical clinical scenario. On one hand, the encouraging results of patch-DNNs have been obtained for the diagnosis of axillary lymph node metastasis in the breast. It is the simplest diagnosis since it yields the two results of positive and negative, which is suitable for computer implementations. On the other hand, the diagnosis of sentinel lymph node metastasis from breast cancer based on FSs has been widely used to aid in the selection of the operation mode in China^18^. Third, the distribution of health resources in China is unbalanced, which the eastern region is obviously more than that in the central and western regions, and there are more diagnosis and treatment patients^19^. Artificial intelligence is needed to assist the related diagnosis and treatment activities. Furthermore, diagnoses with FS may be the most difficult task for pathologists due to the different processes used to form them^20–22^. All of these issues may hinder promoting some operations.

**Figure 1:**
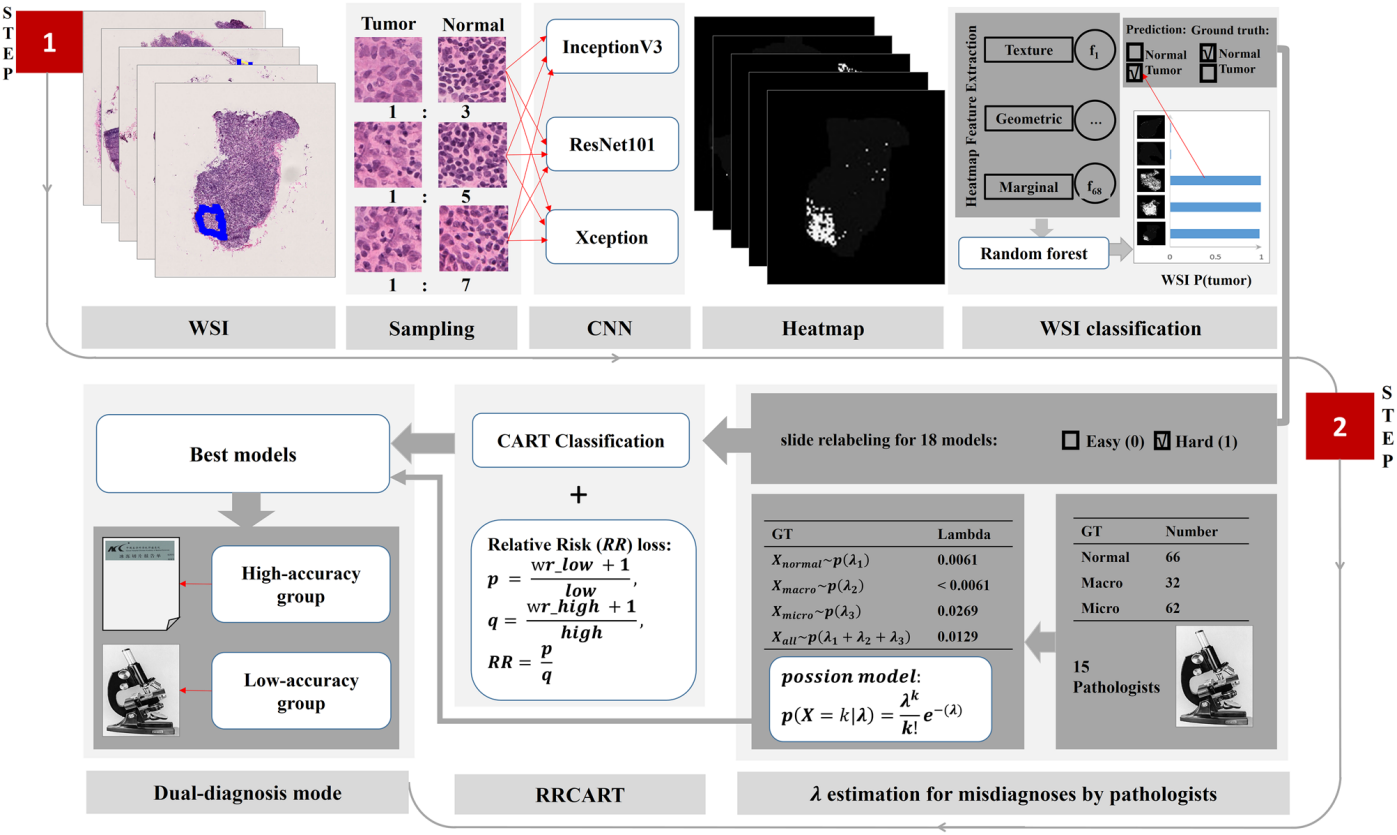
Overview of the Expert-DNN framework presented in this study. See Materials and Methods for complete details. There was two steps in this framework. In step 1, the patch-DNN workflow includes patch and WSI classification. Patches were extracted from tumor and normal regions of WSIs and as input data for DNN training. Patch coordination and probability generated by the model were then used for heatmap construction. A machine learning model (random forest) was trained based on the features extracted from the WSI heatmap and given a WSI-level prediction. The three sampling ratios (1:3, 1:5 and 1:7) correspond to three DNNs (InceptionV3, ResNet101 and Xception), respectively, and the experiment is repeated once. Therefore, we can obtain 18 patch-DNN models. In step 2, a Poisson distribution for the pathology experts and a RRCART were constructed. For the Poisson distribution, the values of λ for the normal, micrometastasis, and macrometastasis cases were estimated based on the incorrect diagnosis frequency of 15 pathology experts for the 160 lymph node FSs. The patch-DNN prediction result was compared with the ground truth to relabel the WSI as either easy (0) or hard (1), corresponding to the prediction result being the same as the ground truth or not. The easy and hard labeling information and the features extracted from the heatmaps were used as the input of the RRCART, and then the high-accuracy group, with an incorrect prediction rate identical to that of the pathology experts, may be ready for pathologists to give diagnostic reports, while the low-accuracy group, with a high frequency of incorrect predictions, must be selected for pathologists to review.

We hypothesized that the identification of misdiagnosis from the patch-DNN would reveal the same accuracy in most sections as the pathologists. First, patch-DNN models were used to diagnose WSIs. Then, all the misdiagnoses from these WSIs were separated by RRCART (Fig. 1). The identification performance was finally evaluated by a Poisson distribution estimated from pathologists’ misdiagnosis data (Fig. 1). Through these processes, the diagnosis report may be finished by the help of a machine-learning algorithm, either recommended for review by a pathologists or ready for pathologists to give diagnostic reports. This method identifies the range of application of the patch-DNN in the diagnosis of lymph node metastasis from breast cancer and therefore may maximally improve the repeatability and efficiency of diagnosis.

## Results

### Influence of the sampling ratio and DNN on the classification metrics

Considering that the sampling ratio and different commonly used DNN architecture may impact the WSI classification metrics, we designed 18 patch-DNN models based on the sampling ratio and patch-DNN method (Fig. 1). We found that the average values of the sensitivity, precision and F1 score of the 18 different models were all above 0.92, while the average values of the specificity, accuracy and AUC were above 0.97. Two-way ANOVA showed that the sampling ratio, DNN architecture and their interaction had no significant effect on the classification metrics (Table S1).

### Misdiagnoses made by patch-DNN models

**Figure 2:**
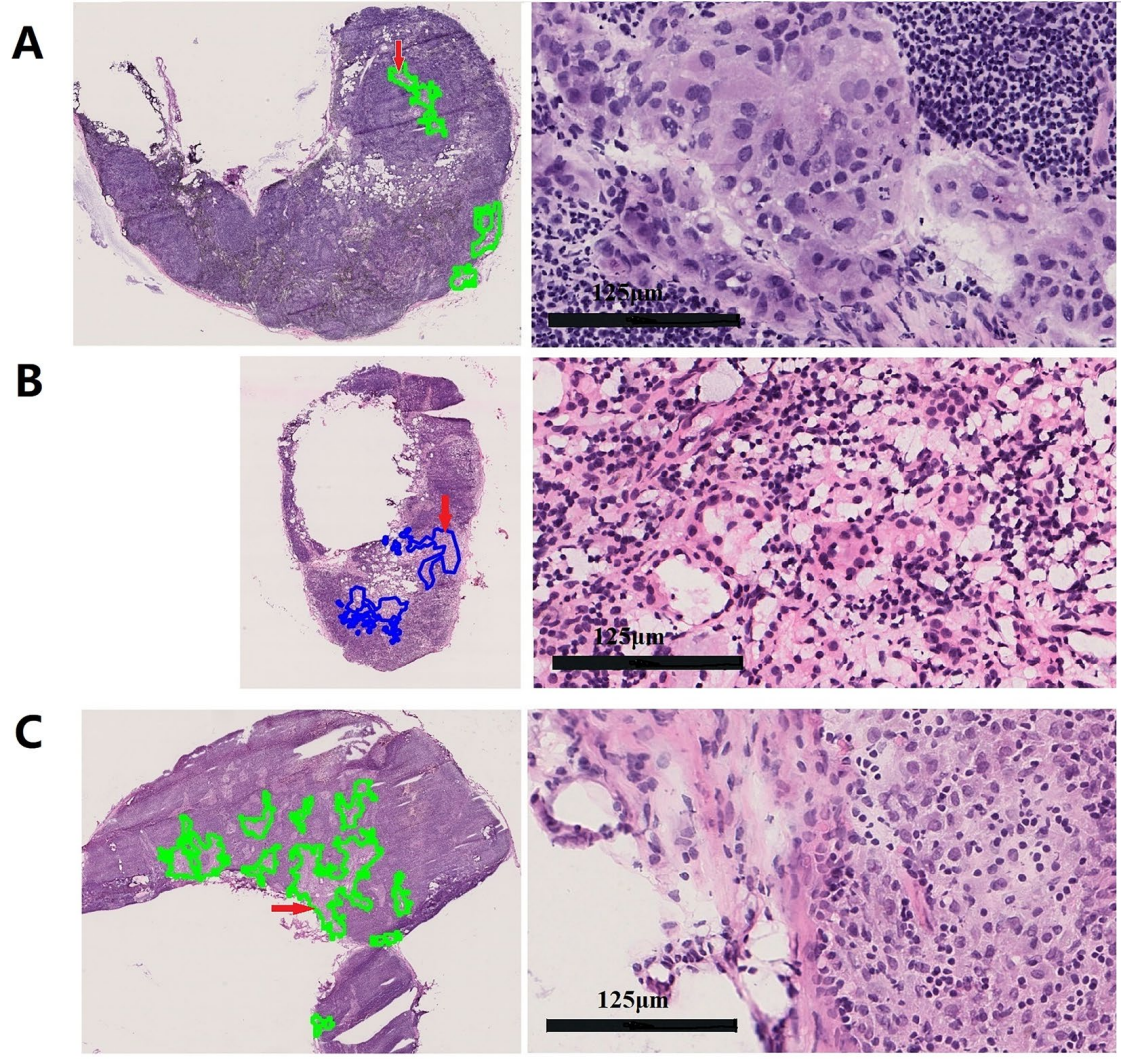
Pathology experts’ analysis of the misclassification errors for the test sets. **A**–**C**, Randomly selected examples of incorrect classification results for the test set. Examples of true positive, false negative and false positive classifications are shown for every WSI, with the figures to the right representing the enlarged areas indicated by the red arrows. (**A**) The true positive area represents an incorrect taken FS during the process of section scanning. (**B**) The false-negative area is the result of cancer tissue growth intermingled with lymphocytes, showing a linear structure that does not gather together. (**C**) The false-positive case, in which the model identifies the section as the medullary cord.

To summarize the statistical characteristics of misdiagnosis among the 18 models, we found the total number of misdiagnosed WSIs was 104, including 60 false-positive and 44 false-negative diagnoses, after the overlapped misdiagnosed samples in the different models were removed. To analyze the reasons for the inconsistency in the diagnoses between these 18 models and the pathological diagnosis of sentinel lymph nodes, the pathology review group reviewed the FSs that were misdiagnosed by the above models. In one FS, the cut edge of a mammary gland was mistaken for a sentinel lymph node, and another model erroneously classified a lymph node without metastasis as a lymph node with metastasis (Fig. 2A). These two WSIs were obtained by taking an incorrect FS during the process of section scanning. In addition, one WSI was difficult to diagnose based only on FSs. In this case, we observed a few suspicious carcinoma cells in the capsule of the lymph node in the FS, while these cells disappeared in the deep section in the paraffin section. Therefore, we could not obtain a definitive diagnosis on this FS. After these three samples were removed, the model made prediction errors from the remaining 101 WSIs, including 41 false negatives (Fig. 2B) and 60 false positives (Fig. 2C).

Notably, 30 WSIs (28.85%) were predicted incorrectly by more than 10 of the 18 models, indicating that the patch-DNN method misclassified certain samples during pathological diagnosis. It will be clinically necessary to refine the patch-DNN to further improve its diagnosis performance.

### Evaluation of the misdiagnosis of the patch-DNN by using a Poisson test

**Figure 3:**
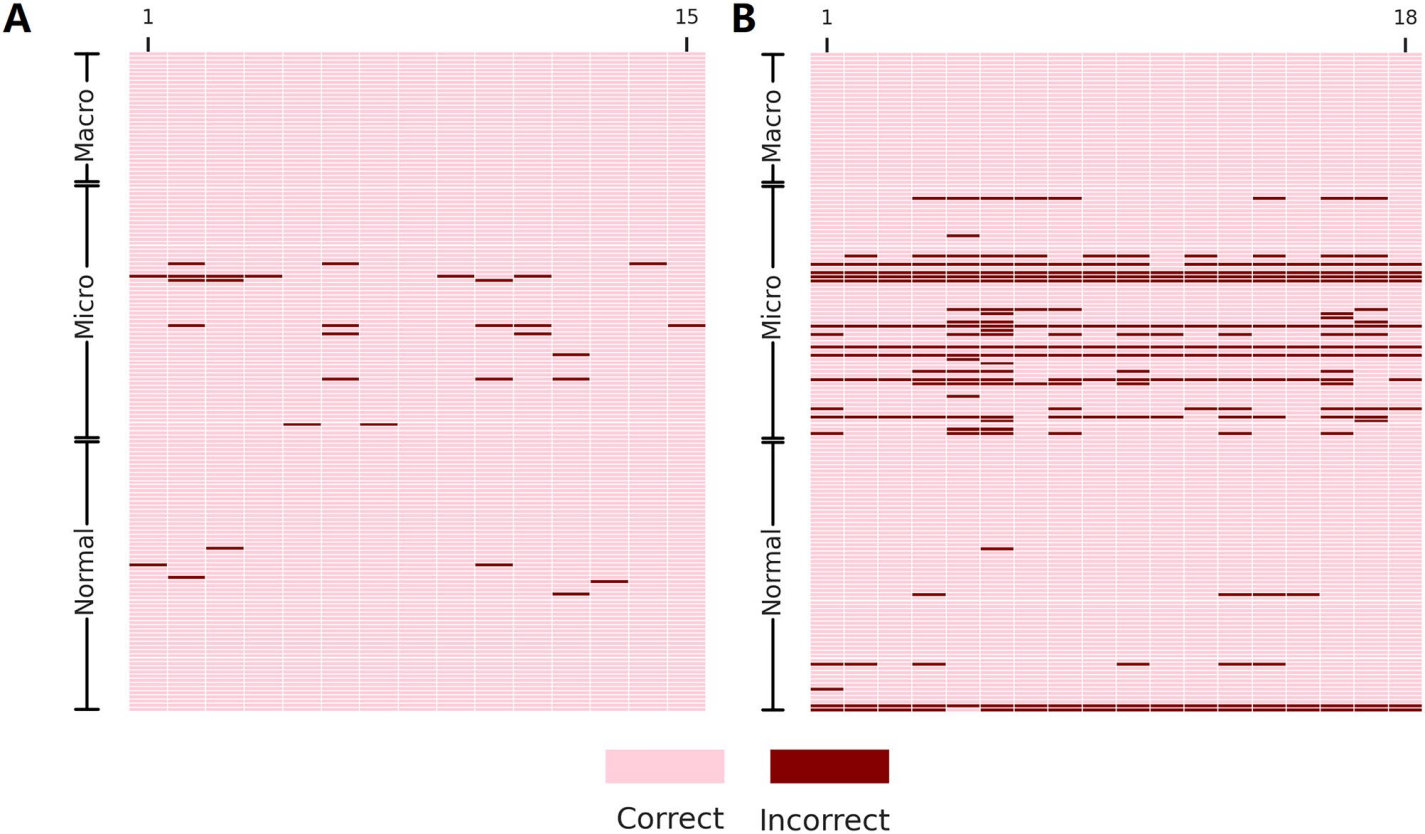
Per-WSI diagnosis for 15 pathology experts (**A**) and 18 patch-DNN models (**(B**). The systematic prediction errors for normal WSIs (> 4) and micrometastasis WSIs (> 6) can clearly be observed in comparison to the experts’ diagnosis. The order of the 18 patch-DNN models is as follows: InceptionV3-1-1:3, InceptionV3-1-1:5, InceptionV3-1-1:7, ResNet101-1-1:3, ResNet101-1-1:5, ResNet101-1-1:7, Xception-1-1:3, Xception-1-1:5, Xception-1-1:7, InceptionV3-2–1:3, InceptionV3-2-1:5, InceptionV3-2-1:7, ResNet101-2-1:3, ResNet101-2-1:5, ResNet101-2-1:7, Xception-2-1:3, Xception-2-1:5, and Xception-2-1:7. The format of the models is the DNN-repeat-tumor normal patch ratio.

To evaluate the performance of the patch-DNN in a statistical manner, we first determined the erroneous diagnosis frequency by comparing the model performance with that of the experienced pathologists. We selected 160 sections from the test set, including 66 normal, 32 macrometastasis and 62 micrometastasis sets (Fig. 1), and then invited 15 experienced pathologists (attending or above doctors with 6–33 years of working experience) from CICAMS to review the FSs manually to establish the Poisson distribution model according to the frequency of incorrect diagnosis (Fig. 3).

We compared the prediction results of the 18 patch-DNN models with the diagnoses of the 15 pathologists (Fig. 3). The results showed that the patch-DNN models produced significantly higher errors for normal and micrometastasis WSIs (*p* < 0.01, Table S2) than those in pathologists. In addition, the five classification evaluation metrics, namely, sensitivity, specificity, precision, accuracy, and F1 score, were significantly different from those of the pathology experts (*p* < 0.01, Table S3). All of the above results suggest that the diagnostic accuracy of the pathologist consensus was higher than the prediction performance of the DNN models.

### High-accuracy predicted WSIs can be separated from low-accuracy WSIs by RRCART

To make patch-DNN models meet the diagnostic requirements for FSs, we divided the prediction results of the patch-DNN models into two categories: high-accuracy WSIs and low-accuracy WSIs (Fig. 1, Supplementary materials).

Based on the above assumption, we labeled each WSI again according to the prediction results of each patch-DNN model and the ground truth (Fig. 1). The number of incorrectly predicted WSIs was far smaller than the number of correctly predicted WSIs as a result of imbalanced data with a ratio of 1:41 on average (Table S4). We designed a classification algorithm combining the relative risk ratio and classification and regression tree (Fig. 1, Supplementary materials). Application of the RRCART algorithm on the test set divided all WSIs into a high-accuracy subset and a low-accuracy subset. A significant difference in misdiagnosis was detected between the results of the original 18 models and the expectations of the pathology experts (*p* < 0.01, Table 1). However, after enrichment by the RRCART algorithm, no significant difference between the expectation of incorrect prediction by the patch-DNN models on the high-accuracy WSIs and that of the pathology experts (*p* > 0.01) was observed. However, for low-accuracy WSIs, a significant difference was found between the number of recognition errors by the 18 models and the expectation of the pathology experts (*p* < 0.01). Significantly, the average proportion of high-accuracy WSIs set to all sets from the 18 models was 83%, and the accuracy for the high-accuracy set reached to 0.995 (average of 4.11 misdiagnosis). Our RRCART method significantly reduced the misdiagnosis rate for 16 patch-DNN models (Table 1, *p* < 0.05, fisher exact test). The above results support that RRCART can help patch-DNN more accurately diagnose most lymph node metastasis from breast cancer.

**Table 1:** Distributions of WSIs incorrectly predicted by patch-DNN models after classification by RRCART.

		Test set		High-accuracy set		Low-accuracy set		
DNN-repeat-tumor normal patch ratio	Ratio (high/total)	Expected incorrectly predicted slides	*P* value	Expected incorrectly predicted slides	*P* value	Expected incorrectly predicted slides	*P* value	Fisher exact test for systematic error between high-accuracy and test set
InceptionV3-1-1:3	0.86	5.62	2.00E-06	4.17	7.86E-01	1.45	3.83E-13	0.003138
InceptionV3-1-1:5	0.81	5.83	9.27E-07	4.01	7.64E-01	1.81	1.28E-12	0.002974
InceptionV3-1-1:7	0.83	5.83	1.25E-04	4.25	9.25E-01	1.58	1.68E-10	0.003535
ReNet101-1-1:3	0.82	5.62	7.23E-06	4.26	7.98E-01	1.36	1.74E-12	0.006282
ReNet101-1-1:5	0.85	5.77	2.77E-09	4.68	6.87E-01	1.09	0.00E + 00	0.014472
ReNet101-1-1:7	0.83	5.73	5.24E-10	4.40	1.57E-01	1.33	0.00E + 00	0.095278
Xception-1-1:3	0.83	5.94	5.00E-05	4.38	4.45E-01	1.56	1.20E-08	0.007071
Xception-1-1:5	0.85	5.77	7.97E-07	4.25	4.19E-01	1.52	9.76E-12	0.009791
Xception-1-1:7	0.84	5.77	3.49E-05	4.43	2.85E-01	1.34	2.12E-08	0.037669
InceptionV3-2-1:3	0.84	5.83	3.45E-06	4.14	5.92E-01	1.70	4.66E-11	0.01299
InceptionV3-2-1:5	0.83	5.89	4.52E-05	4.32	4.34E-01	1.57	1.29E-08	0.036724
InceptionV3-2-1:7	0.83	5.83	1.06E-03	4.50	8.27E-01	1.33	1.81E-08	0.013734
ReNet101-2-1:3	0.81	5.81	3.83E-05	4.40	8.15E-01	1.41	3.69E-11	0.008692
ReNet101-2-1:5	0.83	5.65	2.14E-06	4.29	6.21E-01	1.35	1.72E-12	0.00309
ReNet101-2-1:7	0.81	5.93	3.29E-03	4.18	2.44E-01	1.75	4.63E-04	0.147115
Xception-2-1:3	0.82	5.89	1.37E-05	4.40	6.41E-01	1.49	7.46E-11	0.014449
Xception-2-1:5	0.84	5.81	8.92E-07	4.08	3.88E-01	1.73	5.96E-11	0.022463
Xception-2-1:7	0.81	5.63	2.53E-05	4.44	9.36E-01	1.19	2.54E-13	0.002071

## Discussion

In this study, we focus on the misclassification in DNN algorithms. This is a not well-investigated research area. Here, we hypothesized that the patch-DNN may give the same accuracy in the histopathologic review of lymph node metastasis from breast cancer in most FSs as that of experienced pathologists. To test our hypothesis, we presented a modified classification and regression tree and the Poisson distribution for classifying the risks of misdiagnoses made by common patch-DNNs. We applied the method to 2362 sentinel lymph node FSs collected in CICAMS. Our experiments confirmed the hypothesis and showed the improvement of security issues caused by using DNN in pathologic diagnosis directly and the integration of DNN in a clinical workflow is feasible.

We established a Poisson distribution model according to the frequency of misdiagnosis by pathology experts on 160 FSs. The Poisson distribution can be used to compare the performance of machines and humans with a dataset of thousands of WSIs. Previous studies used the sensitivity, specificity, etc., to compare the best DNN model with the performances of a small number of pathologists^6,12,23,24^ but cannot assess the average performance of different models and cannot compare DNN models and pathologists on datasets that have not been examined by pathology experts. Our results showed that the accuracy of examining FSs by pathology experts was generally higher than that of the 18 models. This result is substantially different from the results in most articles^12,23,24^. The possible reasons for the high degree of homogeneity in the FS diagnosis of sentinel lymph nodes by our pathologists may be as follows: first, the diagnosis of lymph node metastasis is a basic and essential skill for cancer pathologists, as they have been heavily trained on this skill; second, the 5 senior pathologists in this group are all breast cancer specialists and have extensive experience; third, the differences in years of practice may have little effect on the consistency of lymph node diagnosis in the results of our review of 160 randomly chosen FSs.

We used the RRCART algorithm to classify WSIs into high-accuracy and low-accuracy subgroups. The probability of model recognition error of the former is similar to that of the expert review. The latter subgroup requires manual review to meet the diagnostic requirements for FSs. This candidate dual-diagnosis model combining a DNN and experts may reduce the number of required expert diagnosis. Furthermore, we set FSs without negative and macrometastasis prediction errors as filtering thresholds for the models in the high-accuracy subset and retained four qualified DNN models. This process can provide reliable evidence for the choice of operation mode in China. Hence, our approach may improve the diagnosis accuracy of the patch-DNN model.

There were some limitations in our study. First, we used a histopathologic review of lymph node metastasis from breast cancer in FS WSIs to evaluate the accuracy of the patch-DNN. A histopathologic review may not reflect real-world performance. However, this pilot research demonstrated the feasibility of the patch-DNN in histopathologic diagnosis with FSs. Second, we collected 2,362 FSs into this study without considering the clinical features of the patients. In some selected patients, the positive rate of FSs may be higher than in others^25^. However, FS diagnosis is widely used in most hospitals in China without any selection. Our research may reflect the current situation in China. Additionally, Dataset was split on at the WSI level. This means that WSIs from the same patient can be in both training and test set and may result in that classification metrics is overestimated. However, RRCART can make enrichment of the high-accuracy WSIs, reducing the effect on the following clinical workflow. Third, we found no isolated tumor cells in this dataset. However, isolated tumor cells are a rare condition in lymph node metastasis from breast cancer^26^. This may be why no cases are found in some small samples. Finally, we compared the accuracy of the patch-DNN with WSIs and pathologists with traditional glass slides. There are some differences in reading WSIs and glass slides. However, most pathologists are more accustomed to glass slides, and in our group, there were no pathologists who had been trained in reading WSIs. We intended to evaluate the degree of consistency among different pathologists; however, glass-slide reading is closer to the real-world situation for pathologists.

## Conclusion

We employed RRCART and Poisson distribution, using quantitative WSI data to improve the prediction results of the patch-DNNs used for lymph node metastasis detection from breast cancer. Based on the prediction results of DNN and the features extracted at the stage of WSI classification, we can directly execute RRCART and distinguish high-accuracy WSIs from low-accuracy WSIs. In the histopathologic review of lymph node metastasis from breast cancer, our method can be served as an adaptor module to connect the patch-DNN pipeline and the clinical workflow, reducing the security risk of using patch-DNN directly by recommending the low-accuracy WSIs for pathologists to review and the high-accuracy ones for reports.

## Materials and methods

### Images from human subjects

Our research was approved by the Ethics Committee of National Cancer Center/Cancer Hospital, Chinese Academy of Medical Sciences and Peking Union Medical College (20/209-2405). The detailed statement of the approval letter was provided in the Related files. Briefly, in July 6, 2020, the Ethics Committee received the application for approval of the clinical research plan—computer recognition strategy for lymph node metastasis of breast cancer and the application for exemption from informed consent. This met the requirements of rapid review according to the ethics committee SOP, the ethics committee held a serious discussion and voted in July 8, 2020. The number of voters was 2, and agreed: 2 votes, against: 0 votes. Approval result: agree, agree to carry out the study. In this part of the series, patient consent was not required, as no risk to the participants was anticipated. The authors confirm that all methods were carried out in accordance with relevant guidelines and regulations.

We collected FSs of sentinel lymph nodes from breast cancer patients from the Department of Pathology, Cancer Institute & Hospital, Chinese Academy of Medical Sciences (CICAMS), China, from January 2017 to November 2019. The 8–10 µm FSs were hematoxylin and eosin-stained. The final dataset consisted of 2362 patient-exclusive WSIs (129 micrometastases, 353 macrometastases and 1879 normal). All the information on the 499 patients and WSIs was deidentified. We scanned the glass slides containing these sections with a Nano Zoomer S210 scanner at 40X equivalent magnification. All slides were scanned at a resolution of 0.243 μm per pixel.

### Hardware and software

All of our experiments were implemented on three servers, each containing two NVIDIA Tesla P40 Graphic Processing Unit (GPU) cards. We used OpenSlide^27^ Python (version 1.1.1) to read the WSI files and TensorFlow^28^ (version 1.8.0) to load patch data, train the models and infer the WSIs. Finally, we obtained the receiver operating characteristic (ROC) curve using scikit-learn^29^ (version 0.23), statistical analysis results from SPSS (version 22.0), and plots from Seaborn (version 0.9.0) and matplotlib (version 3.2.0).

### Reference standard

We followed the method of Bejnordi et al.^6^ to perform annotation with some small modifications. In brief, all metastatic lesions from positive FS WSIs were first annotated manually by 2 residents with 2–3 years of work experience using the open-source tool ASAP 1.8 (https://github.com/computationalpathologygroup/ASAP/releases), and then each annotated WSI was reviewed in detail by a breast subspecialist pathology review group composed of 3 pathology experts (attending doctor and above) with a mean working experience of 14.3 years (9–18 years). These reviewing reports of the FSs by the pathology expert group were used as the ground truth. The type of lymph node metastasis was divided into three groups: micrometastasis, macrometastasis and negative, according to the American Joint Committee on Cancer (AJCC 8th)^30^.

### Dataset splitting

The dataset was divided into training, validation and test subsets at the WSI level with repeated random separation until no significance (*P* > 0.10) was detected between any two subsets with respect to all features (Table S5). The *P* value was calculated using a t-test for continuous features and Fisher’s exact test for the categorical features. All the features were extracted from the tissue contours of both positive and negative WSIs and metastatic lesion contours from only positive WSIs.

To explore the relationship between the number of annotated WSIs and the saturation of the classification metrics including the sensitivity, specificity, precision, accuracy, F1 score and AUC, 2362 FSs were randomly divided at the WSI level for training with size serials (100, 300, 600, 1000, 1500), with 202 FSs used for validation and 630 for testing (Table S6). In the training group, each previous training dataset became a subset of the following datasets. The whole process was randomly repeated 5 times. Our results showed that when the number of training WSIs reached 600, all six metrics began to saturate (Fig. S1A–F). Therefore, 600 WSIs for the training sets was the minimum number required to saturate the above classification evaluation metrics. Unless otherwise stated, 600 WSIs were used in the following model training (Table S6).

For the task of influence of the sampling ratio and DNN on the classification metrics, the FS WSIs were randomly divided into the training, validation and test subset with their sizes fixed at 600, 202 and 1560, respectively (Table S6). For validation of the RRCART algorithm task, the 1560 test subset above was further split into training and test set with sizes fixed at 780 and 780, respectively.

### Patch sampling

Patches for DNN training were generated by sampling from the training WSIs. First, due to the large size of the WSIs, we used Otsu’s method^31^ to efficiently discard all background and fat patches in each WSI. Second, to avoid sampling biases, we designed a random-sampling strategy according to the following: (1) Each WSI with or without metastatic cancer was selected with equal probability. (2) For each region of interest in one WSI, we collected enough patches from each region of interest with random coordinates generated from a uniform distribution to form a candidate patch set of the WSI. (3) Normal and tumor patches with fixed numbers were randomly selected from the patch-set for each WSI. The fixed number can be calculated easily according to the tumor-normal patch ratio. Previous work has reported that increasing the normal to tumor patch ratio could reduce the false positives^32^, in our work, we tentatively set the ratio of tumor to normal patches at 1:3, 1:5, and 1:7.

### Patch-DNN method for frozen-section diagnosis

Our patch-DNN method consists of a patch-level classification and a WSI-level classification. At the patch-level classification stage, a random-sampling method was used to extract 256 × 256 patches from the WSI training set. The patch-based classifier was trained to estimate the class of each patch. Then, we partitioned each WSI into 256 × 256 patches without overlapping. The probability values of the patches, predicted by the classifier in the first stage, were embedded into a heatmap image.

At the WSI-level classification stage, the corresponding heatmaps were used as the basic data for the following postprocessing method to discriminate the classes of WSIs. We extracted several features (Table S5) from heatmaps to train a WSI-based classification model, and a random forest was trained as the WSI-based classifier.

We used three sampling ratios (1:3, 1:5, and 1:7) corresponding to three commonly used DNNs (InceptionV3, ResNet101 and Xception) and repeated patch sampling and DNN training twice to construct a total of 18 patch-DNN models to study the influence of the sampling ratio and DNN on the classification metrics. Considering the long-term cost (2 months for 18 models) and the small difference between the results of the two repeated experiments, we did not repeat the experiment more times.

### DNN model training and validation

The training workflow of the DNN model is as follows. We trained the DNNs with the stochastic gradient descent optimizer in TensorFlow (version 1.8.0)^28^. The initial learning state was 0.01, and the decay rate was 0.1. All models were initialized with the pre-trained weights on ImageNet (https://github.com/tensorflow/), and cross entropy was used as the loss function to update the network parameters. To test the stability and generalization of the system framework, we separately selected InceptionV3, Xception71, and ResNet101 as our backbone CNN model, all of which were trained on patches with 256 × 256 pixels (~ 0.22 μm/pixel) at 40X magnification from the training WSIs. Then, based on the validation set, we completed the training of the DNN model within 75 epochs (approximately 72 h) and without overfitting.

### Classification metrics

Six general statistical classification metrics, including sensitivity, specificity, precision, accuracy, F1 score (Eq. 1) and AUC value, were used to estimate the performances of the classifiers. The misdiagnosis rate is equal to (1-accuracy). The AUC value was obtained by using scikit-learn^29^ (version 0.23).

1 $${\text{F1}}\;{\text{ score }} = \, ({2} \times {\text{precision}} \times {\text{sensitivity}}) \, / \, \left( {{\text{precision}} + {\text{sensitivity}}} \right)$$

### Incorrect diagnosis rate of FSs by pathology experts

We hypothesized that no systematic errors (misdiagnosis made by most of the experts or the models) would be made by the expert group in the diagnosis of 160 FSs of sentinel lymph nodes (Fig. 1). We also hypothesize that pathology experts and model diagnosis (prediction) of a certain number of WSIs obey the Poisson distribution (Eq. (2)).

2 $$p\,(X=k|\lambda )=\frac{{\lambda }^{k}}{k!}{e}^{-\lambda }$$

Incorrect diagnosis rates for pathology experts were scaled to per WSI per examination. The incorrect diagnosis rate (λ_0_) was calculated by adding up the number of incorrect diagnosis of the 15 pathology experts for all 160 WSIs randomly selected from the test set and then dividing by the total number of diagnosis (160 * 15).

The *P* value was calculated as:

3 $$p\;{\text{value}} = 1 - \sum\limits_{{k = 0}}^{{x - 1}} {\frac{{e^{{ - \lambda }} \lambda ^{k} }}{{k!}}}$$

where x is the observed number of incorrect diagnoses (predictions) for a certain number of WSIs (n) and λ is calculated as n*λ_0_. For example, 228 incorrect diagnoses were made by the 18 patch-DNN models and 25 incorrect diagnoses by the 15 experts for 62 micrometastasis WSIs. We calculated the probability of micrometastasis as [1-Poisson cumulative distribution function (228-1, 62 * 18 * λ_0-micro_)], where λ_0-micro_ reflects the incorrect diagnosis rate for the 15 pathology experts examining the 62 micrometastasis WSIs and was calculated as [25/(62 * 15)].

### Relative risk classification and regression tree algorithm

We hypothesized that the prediction results of the patch-DNN models could be divided into two categories: high-accuracy WSIs, with a frequency of model recognition errors similar to that of pathology experts, and low-accuracy WSIs, with a significantly higher frequency of model recognition errors than that of experts.

The probability $p$ is defined as the ratio of the number of incorrectly predicted WSIs (abbreviation, *S*_*wr_low*_) to the total number of predicted WSIs (abbreviation, *S*_*low*_) with low accuracy; variable $q$ is defined as the ratio of the number of incorrectly predicted WSIs (abbreviation, *S*_*wr_high*_) to the total number of predicted WSIs (abbreviation, *S*_*high*_) with high accuracy. By this definition, we can easily deduced *S*_*wr_low*_ + *S*_*wr_high*_ is equal to the number of the hard WSIs, corresponding to the prediction result different from the ground truth. We add 1 to the numerator of $p$ and $q$ to prevent the number of incorrectly diagnosed WSIs from equaling to zero, which increases the computational robustness of the WSI diagnosis results. Their calculation methods are given in Eqs. (4–6). As a matter of fact, the set *S*_*high*_ is always larger than *S*_*high*_ due to our proposed classification model, so we design the hyper parameter *high_per* given in Eq. (7).

4 $$p=\frac{{S}_{wr\_low}+1}{{S}_{low}+1}$$

5 $$q=\frac{{S}_{wr\_high}+1}{{S}_{high}+1}$$

6 $$RR=\frac{p}{q}$$

7 $$high\_per=\frac{{S}_{high}}{{S}_{low}}$$

We implemented the cost-sensitive learning process^33^ by combining a RR index and a classification and regression tree (CART)^34^. The RR index is the ratio of the incorrect diagnosis probability between the low- and high-accuracy WSI sets and is designed as the loss function for the training of the RRCART, which mainly implies the risk of incorrect prediction for WSIs with low accuracy relative to that of WSIs with high accuracy. The algorithm below shows how to replace the Gini method with our relative risk method.

Algorithm 1 describes the stop conditions for the recursive method, which is also used in a CART classification tree, and algorithm 2 shows how to replace the Gini method with our relative risk method (Supplementary materials).

### Statistical analysis

The Poisson distribution, Fisher’s exact test and t-test analyses were performed using R statistical software version 3.6.1^35^. Two-way repeated measures analysis of variance (ANOVA)^36^ was performed by SPSS 22.0 (International Business Machine (IBM), Armonk, USA).

## Supplementary Information

Supplementary Information 1.

Supplementary Information 2.

Supplementary Information 3.

Supplementary Information 4.

Supplementary Information 5.

Supplementary Information 6.

Supplementary Information 7.

Supplementary Information 8.

## Data Availability

The data are not publicly available due to hospital regulations. But data requests with aims will be needed to assess the reasonability. After approval from the hospital and the corresponding authors, de-identified clinical data will be provided.

## Code availability

The source code for this work can be downloaded from https://gitee.com/chencancan1018/dch-lymph-node-module.
