# Supplementary figures and images for: Identification of misdiagnosis by deep neural networks on a histopathologic review of breast cancer lymph node metastases
Source: Sci Rep. 2022 Aug 5;12:13482. doi: 10.1038/s41598-022-17606-0 (PMC9355979; doi:10.1038/s41598-022-17606-0)

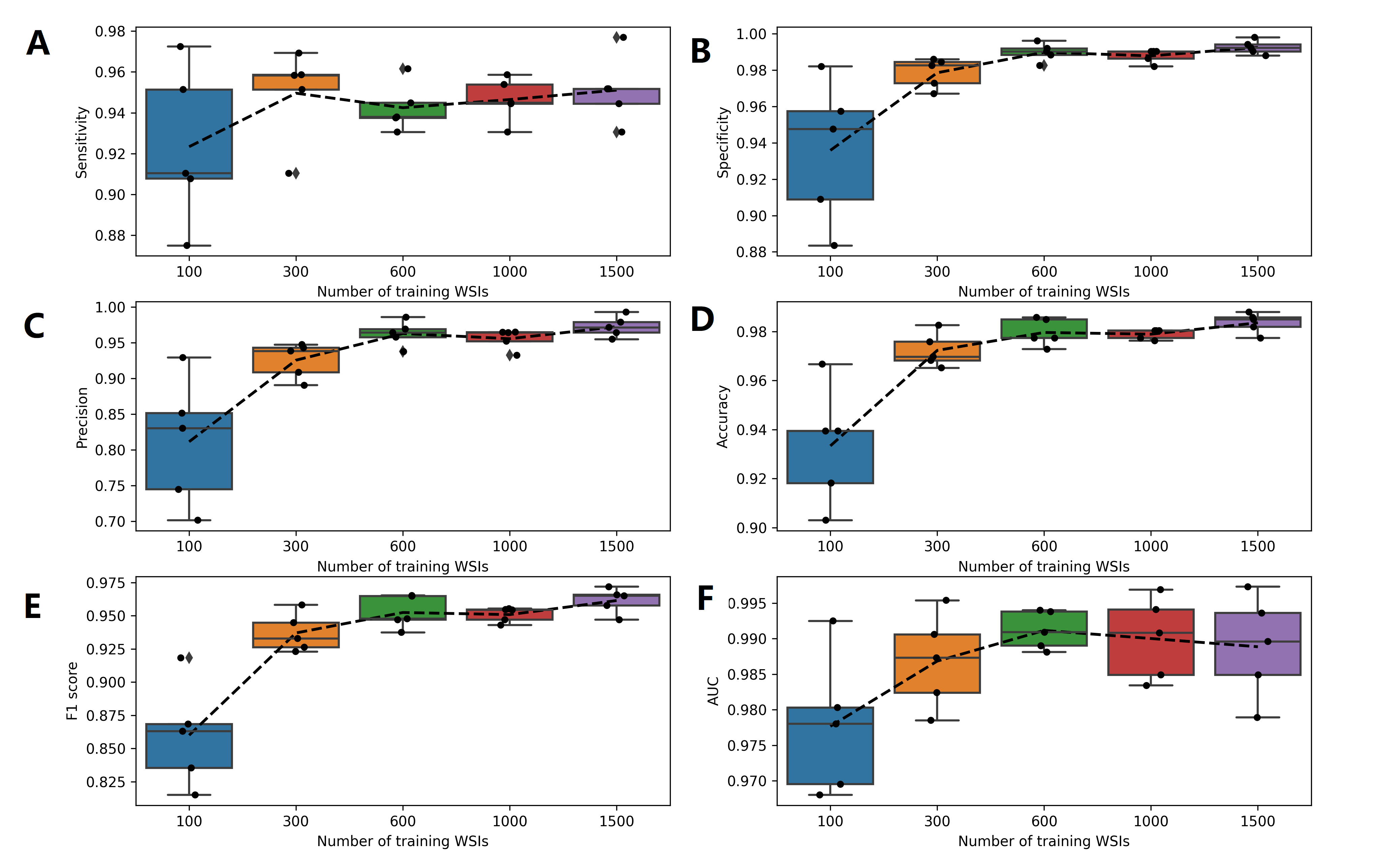

Supplement: Supplementary file 1 — Supplementary Information 1. [file 41598_2022_17606_MOESM1_ESM.tiff]
