# Supplementary material for: Identification of misdiagnosis by deep neural networks on a histopathologic review of breast cancer lymph node metastases
Source: Sci Rep. 2022 Aug 5;12:13482. doi: 10.1038/s41598-022-17606-0 (PMC9355979; doi:10.1038/s41598-022-17606-0)
Supplement: Supplementary file 2 — Supplementary Information 2. [file 41598_2022_17606_MOESM2_ESM.docx]

**Supplementary materials**

1. **Supplementary Figure legend**

**Figure S1 Data size plays an important role in achieving stable status for patch-DNN classification performance.** Training of InceptionV3 was performed with datasets of increased size. For every reported training set size, five models were trained, and the six classification metrics, including sensitivity (A), specificity (B), precision (C), accuracy (D), F1 score (E) and AUC (F) are calculated on the test set and reported as box plots (*n* = 5). This experiment underlies the fact that 600 is the minimum number of WSIs which are necessary for generalization of stable classification performance.

**Figure S2 Approval Letter of Ethic Committee**

1. **Our RRCART algorithm**

Algorithm 1 describes the stop conditions for the recursive method, which is also used in a CART classification tree, and algorithm 2 shows how to replace the Gini method with our relative risk method (Supplementary materials).

**Algorithm 1:** Relative risk classification and regression tree

**Input**: Training set ;


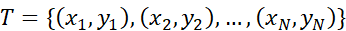

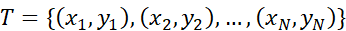

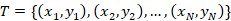


Features set ;


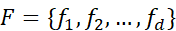

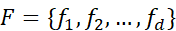

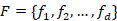


Min samples *k*;

Ending condition ;


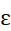

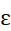

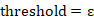


**Process**: TreeGenerate()


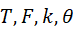

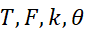

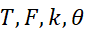


(1). Generate node;

(2). **If**  **for** all samples in that have the same category, **then**


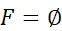

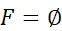

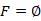

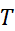

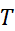


(3). Marked as a leaf node; **return**

(4). **end if**

(5). **If** , **then**


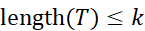

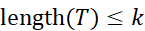

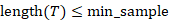


(6). Marked as a leaf node; **return**

(7). **end if**

(8). Select the optimal feature, and compute and *RR* by Algorithm 2 (SelectFeature(*T, F*));


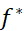

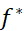

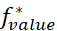

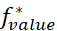

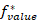


(9). is split into and due to the optimal features and ;


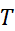

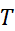

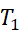

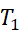

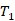

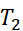

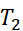

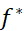

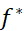

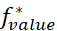

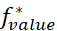

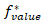


(10). Generate two branch nodes;

(11). **If** length for list [y in T_1_ if y == 1] ≥ length for list [y in T2 if y==1], **then**


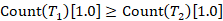


(12). and ;


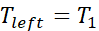

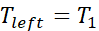

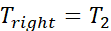

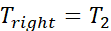

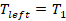

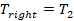

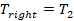


(13). **else**

(14). and ;


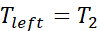

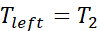

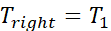

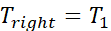

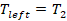

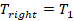

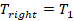


(15). **If** , or is empty, or , **then**


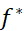

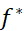

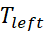

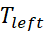

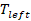

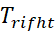

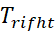

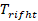

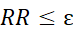

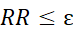


(16). The branch node is marked as a leaf node; **return**

(17). **else**

(18). Mark TreeGenerate(,*F*\{},,) and TreeGenerate(,*F*\{},,) as branch nodes


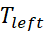

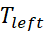

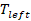

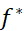

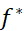

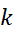

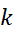

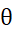

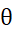

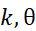

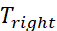

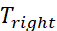

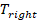

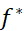

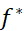

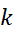

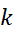

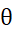

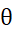


(19). **end if**

**Output**: Relative risk decision tree

**Algorithm 2**: Select the optimal splitting points for features

**Input**: Training set ;


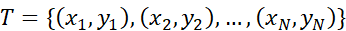

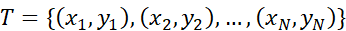


Features set ;


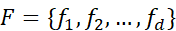

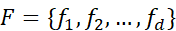


Threshold and (<).


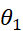

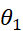

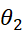

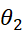

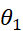

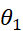

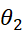

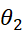


**Process**: SelectFeature(*T, F*)

(1). Compute *RR*() and high-per in equation (10) and (11)；


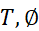

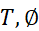


(2). Set =, , and ;


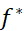

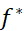

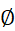

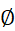

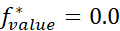


(3). **for**, **do**

(4). **for** each of feature in training set *T*, **do**

(5). Split set *T* into a low-confidence set and high-confidence set by , marked as

1. ;

2.

(6). Compute *RR*(,) and *high_per* by equation (10-11).

(7). **If ( and**

**and** ), **then**

(8).; ; ;

(9). **end if**

(10). **end for**

(11). **end for**

**Output**:

Note that and represented the selected optimal feature set and the corresponding feature value, and we tried all hyper parameters based on our dataset, and the details could be found in our public code.
