# Supplementary material for: Identification of misdiagnosis by deep neural networks on a histopathologic review of breast cancer lymph node metastases
Source: Sci Rep. 2022 Aug 5;12:13482. doi: 10.1038/s41598-022-17606-0 (PMC9355979; doi:10.1038/s41598-022-17606-0)
Supplement: Supplementary file 3 — Supplementary Information 3. [file 41598_2022_17606_MOESM3_ESM.docx]

**Table S1 Application of two-way repeated measures ANOVA to determine the classification metrics performance for 3 DNNs with different patch ratios**

| **score** | **source** | **df** | **mean square** | **F** | **significance** |
| --- | --- | --- | --- | --- | --- |
| sensitivity | model | 2 | 0.002 | 31.000 | 0.031 |
|  | sampling | 2 | 0.000 | 0.420 | 0.704 |
|  | model * sampling | 4 | 0.001 | 2.424 | 0.206 |
| specificity | model | 2 | 0.000 | 2.333 | 0.300 |
|  | sampling | 2 | 0.000 | 2.301 | 0.303 |
|  | model * sampling | 4 | 0.000 | 0.635 | 0.665 |
| precision | model | 2 | 0.000 | 2.146 | 0.318 |
|  | sampling | 2 | 0.000 | 2.753 | 0.266 |
|  | model * sampling | 4 | 0.000 | 0.757 | 0.603 |
| Accuracy | model | 2 | 0.000 | 15.551 | 0.060 |
|  | sampling | 2 | 0.000 | 1.079 | 0.481 |
|  | model * sampling | 4 | 0.000 | 6.032 | 0.055 |
| F1 score | model | 2 | 0.001 | 18.069 | 0.052 |
|  | sampling | 2 | 0.000 | 0.868 | 0.535 |
|  | model * sampling | 4 | 0.000 | 5.052 | 0.073 |
| AUC | model | 2 | 0.000 | 4.912 | 0.169 |
|  | sampling | 2 | 0.000 | 0.010 | 0.990 |
|  | model * sampling | 4 | 0.000 | 5.114 | 0.072 |
