# Supplementary material for: Identification of misdiagnosis by deep neural networks on a histopathologic review of breast cancer lymph node metastases
Source: Sci Rep. 2022 Aug 5;12:13482. doi: 10.1038/s41598-022-17606-0 (PMC9355979; doi:10.1038/s41598-022-17606-0)
Supplement: Supplementary file 4 — Supplementary Information 4. [file 41598_2022_17606_MOESM4_ESM.docx]

**Table S2 Comparison between the prediction results of 18 patch-DNN models and the diagnoses of 15 experts**

| Category | λ for pathology experts | Observed events for pathology experts | λ for patch-DNN | Observed events for patch-DNN | Expected events for patch-DNN | Significance* |
| --- | --- | --- | --- | --- | --- | --- |
| All | 0.0129 | 31 | 0.9549 | 275 | 27.2 | 0 |
| Normal | 0.0061 | 6 | 0.0397 | 47 | 7.2 | 0 |
| Micro | 0.0269 | 25 | 0.2043 | 228 | 30 | 0 |

***α= 0.01**
