# Supplementary material for: Identification of misdiagnosis by deep neural networks on a histopathologic review of breast cancer lymph node metastases
Source: Sci Rep. 2022 Aug 5;12:13482. doi: 10.1038/s41598-022-17606-0 (PMC9355979; doi:10.1038/s41598-022-17606-0)
Supplement: Supplementary file 5 — Supplementary Information 5. [file 41598_2022_17606_MOESM5_ESM.docx]

**Table S3 Sensitivity, specificity, precision, accuracy and F1 score for the diagnosis of 160 WSIs of 15 pathologists and 18 patch-DNN models**

| *Test for homogeneity of variance* | | | T-Test | | |
| --- | --- | --- | --- | --- | --- |
| *score* | *F* | *significance* | *T* | *df* | *Significance*  *,two-tails* |
| sensitivity | 9.650 | 0.004 | 10.757 | 20.923 | 0.000 |
| specificity | 7.660 | 0.009 | 8.754 | 27.310 | 0.000 |
| precision | 10.395 | 0.003 | 9.377 | 26.449 | 0.000 |
| accuracy | 8.726 | 0.006 | 12.865 | 22.256 | 0.000 |
| F1 | 9.234 | 0.005 | 11.940 | 21.011 | 0.000 |
