# Supplementary material for: Identification of misdiagnosis by deep neural networks on a histopathologic review of breast cancer lymph node metastases
Source: Sci Rep. 2022 Aug 5;12:13482. doi: 10.1038/s41598-022-17606-0 (PMC9355979; doi:10.1038/s41598-022-17606-0)
Supplement: Supplementary file 6 — Supplementary Information 6. [file 41598_2022_17606_MOESM6_ESM.docx]

**Table S4 Statistics of WSIs incorrectly predicted by patch-DNN models for RRCART**

| DNN-repeat-tumor normal patch ratio | Number of Incorrect slides in training set for RRCART | Number of Incorrect slides in test set for RRCART | Number of incorrectly predicted slides by patch-DNN | Number of correctly predicted slides by patch-DNN |
| --- | --- | --- | --- | --- |
| InceptionV3-1-1:3 | 21 | 20 | 41 | 1519 |
| InceptionV3-1-1:5 | 22 | 21 | 43 | 1517 |
| InceptionV3-1-1:7 | 16 | 17 | 33 | 1527 |
| ReNet101-1-1:3 | 19 | 19 | 38 | 1522 |
| ReNet101-1-1:5 | 25 | 25 | 50 | 1510 |
| ReNet101-1-1:7 | 26 | 26 | 52 | 1508 |
| Xception-1-1:3 | 18 | 18 | 36 | 1524 |
| Xception-1-1:5 | 20 | 21 | 41 | 1519 |
| Xception-1-1:7 | 19 | 18 | 37 | 1523 |
| InceptionV3-2-1:3 | 20 | 20 | 40 | 1520 |
| InceptionV3-2-1:5 | 17 | 18 | 35 | 1525 |
| InceptionV3-2-1:7 | 16 | 15 | 31 | 1529 |
| ReNet101-2-1:3 | 18 | 18 | 36 | 1524 |
| ReNet101-2-1:5 | 20 | 20 | 40 | 1520 |
| ReNet101-2-1:7 | 14 | 14 | 28 | 1532 |
| Xception-2-1:3 | 20 | 19 | 39 | 1521 |
| Xception-2-1:5 | 22 | 21 | 43 | 1517 |
| Xception-2-1:7 | 18 | 18 | 36 | 1524 |
