# Supplementary material for: Identification of misdiagnosis by deep neural networks on a histopathologic review of breast cancer lymph node metastases
Source: Sci Rep. 2022 Aug 5;12:13482. doi: 10.1038/s41598-022-17606-0 (PMC9355979; doi:10.1038/s41598-022-17606-0)
Supplement: Supplementary file 7 — Supplementary Information 7. [file 41598_2022_17606_MOESM7_ESM.docx]

**Table S5. Extracted Features**

| Category | Feature name | Description |
| --- | --- | --- |
| Geometric features | AreaMax, AreaMin, AreaMean, AreaSTD, AreaVar, AreaSkew, AreaKurt | The maximum, minimum, mean area of all connected regions; the standard deviation, variance, skewness, kurtosis of area of all connected region |
|  | PMMax, PMMin, PMMean, PMSTD, PMVar, PMSkew, PMKurt | The maximum, minimum, mean perimeter of all connected regions; the standard deviation, variance, skewness, kurtosis of perimeter of all connected region |
|  | DMMax, DMMin, DMMean, DMSTD, DMVar, DMSkew, DMKurt | The maximum, minimum, mean length of the major axis of all connected regions; the standard deviation, variance, skewness, kurtosis of the major axis of all connected region |
|  | ECCTMax, ECCTMin, ECCTMean, ECCTSTD, ECCTVar, ECCTSkew, ECCTKurt | The maximum, minimum, mean eccentricity of all connected regions; the standard deviation, variance, skewness, kurtosis of eccentricity of all connected region |
|  | ExtentMax, ExtentMin, ExtentMean, ExtentSTD, ExtentVar, ExtentSkew, ExtentKurt | The maximum, minimum, mean extent (ratio of pixels in the region to pixels in the total bounding box) of all connected regions; the standard deviation, variance, skewness, kurtosis of extent of all connected region |
|  | SolidityMax, SolidityMin, SoliditySTD, SolidityMean, SolidityVar, SoliditySkew, SolidityKurt | The maximum, minimum, mean solidity (ratio of pixels in the region to pixels in the convex hull image) of all connected regions; the standard deviation, variance, skewness, kurtosis of solidity of all connected region |
| Texture features | GradMax, GradMin, GradMean, GradSTD, GradVar, GradSkew, GradKurt, GradEntropy, GradEnergy, GradContrast, GradDissimilarity, GradHomogeneity, GradCorrelation | The maximum, minimum, mean, standard deviation, variance, skewness, kurtosis of the gradient channel value; the entropy, sum of squared elements, contrast, dissimilarity, homogeneity, correlation of the normalized co‑occurrence matrix of gradient channel value |
|  | IntensityMax, IntensityMin, IntensityMean, IntensitySTD, IntensityVar, IntensitySkew, IntensityKurt, IntensityEntropy, IntensityEnergy, IntensityContrast, IntensityDissimilary, IntensityHomogeneity, IntensityCorrelation | The maximum, minimum, mean, standard deviation, variance, skewness, kurtosis of the raw pixel value; the entropy, sum of squared elements, contrast, dissimilarity, homogeneity, correlation of the normalized co‑occurrence matrix of raw pixel value |
| Marginal features | CannyNonzero, CannyMean | Number of pixels with nonzero canny value; the mean of canny value |
| Other features | RegionCount | The number of the connected regions |
|  | RatioTT | The ratio of the tumor connected region to the tissue region |
|  | LTArea | The area of the largest connected region |
|  | LTMajorAxis | The major axis of the largest connected region |
|  | PixelsCount | The number of pixels with predicted probability larger than 0.9 |
|  | AvgPred | The average value of pixels in the tumor connected region |
