# Supplementary material for: Identification of misdiagnosis by deep neural networks on a histopathologic review of breast cancer lymph node metastases
Source: Sci Rep. 2022 Aug 5;12:13482. doi: 10.1038/s41598-022-17606-0 (PMC9355979; doi:10.1038/s41598-022-17606-0)
Supplement: Supplementary file 8 — Supplementary Information 8. [file 41598_2022_17606_MOESM8_ESM.docx]

**Table S6 Training, validation and test settings for evaluating the data size dependence of classification and the performance of WSI classification comparison among the 18 models**

| Task | Dataset | Number of tumor slides | Number of normal slides | Total |
| --- | --- | --- | --- | --- |
| Data size dependence | Training set1 | 50 | 50 | 100 |
|  | Training set2 | 100 | 200 | 300 |
|  | Training set3 | 150 | 450 | 600 |
|  | Training set4 | 200 | 800 | 1000 |
|  | Training set5 | 250 | 1250 | 1500 |
|  | Validation set | 37 | 165 | 202 |
|  | Test set | 130 | 530 | 660 |
| Performance evaluation | Training | 150 | 450 | 600 |
|  | Validation | 37 | 165 | 202 |
|  | Test | 294 | 1266 | 1560 |
